# Supplementary figures and images for: Construction and validation of a PANoptosis-related lncRNA signature for predicting prognosis and targeted drug response in thyroid cancer
Source: PeerJ. 2023 Sep 1;11:e15884. doi: 10.7717/peerj.15884 (PMC10476615; doi:10.7717/peerj.15884)

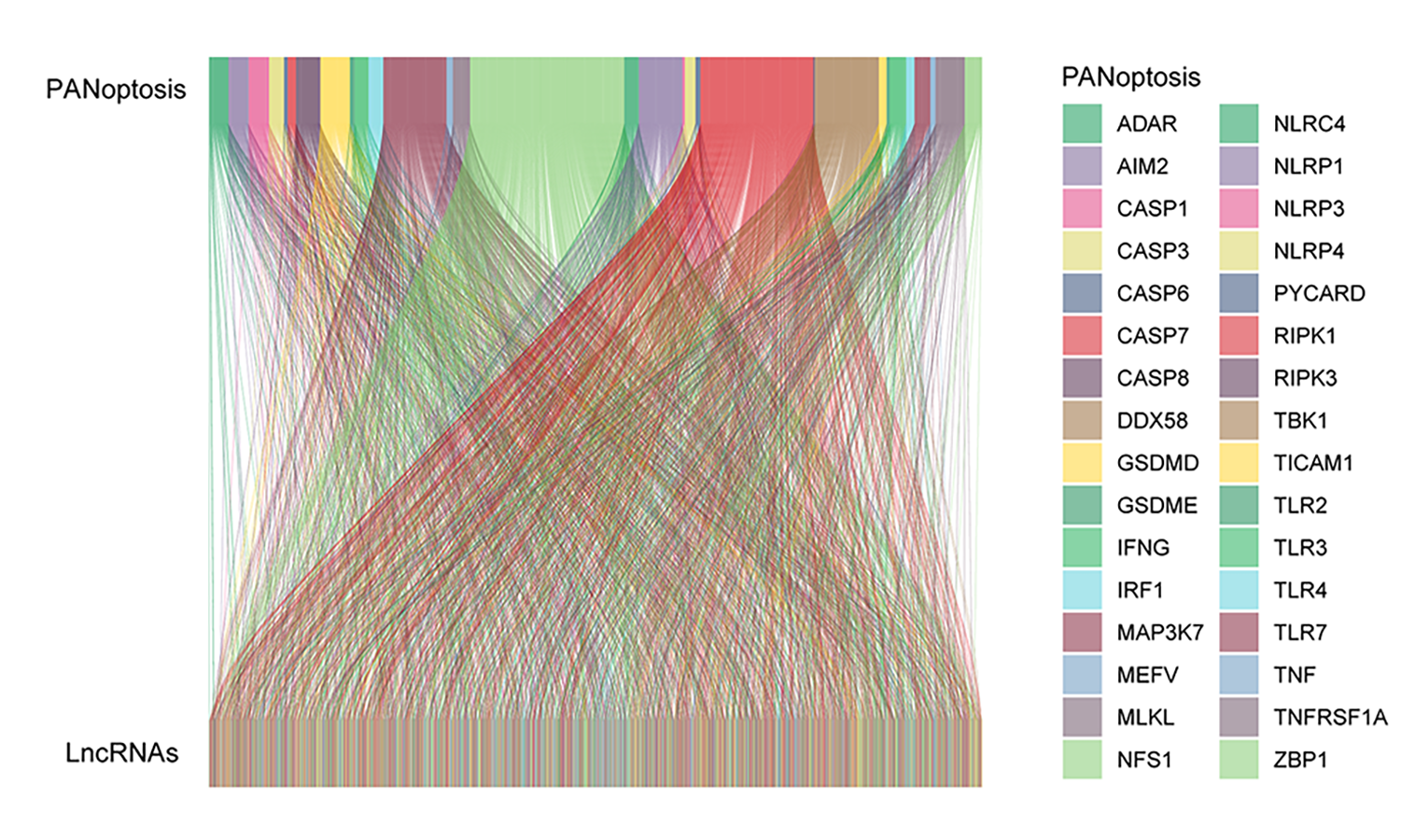

Supplement: Supplemental Information 1 [file peerj-11-15884-s001.png]

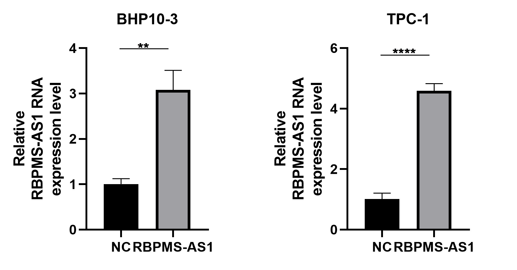

Supplement: Supplemental Information 2 [file peerj-11-15884-s002.png]

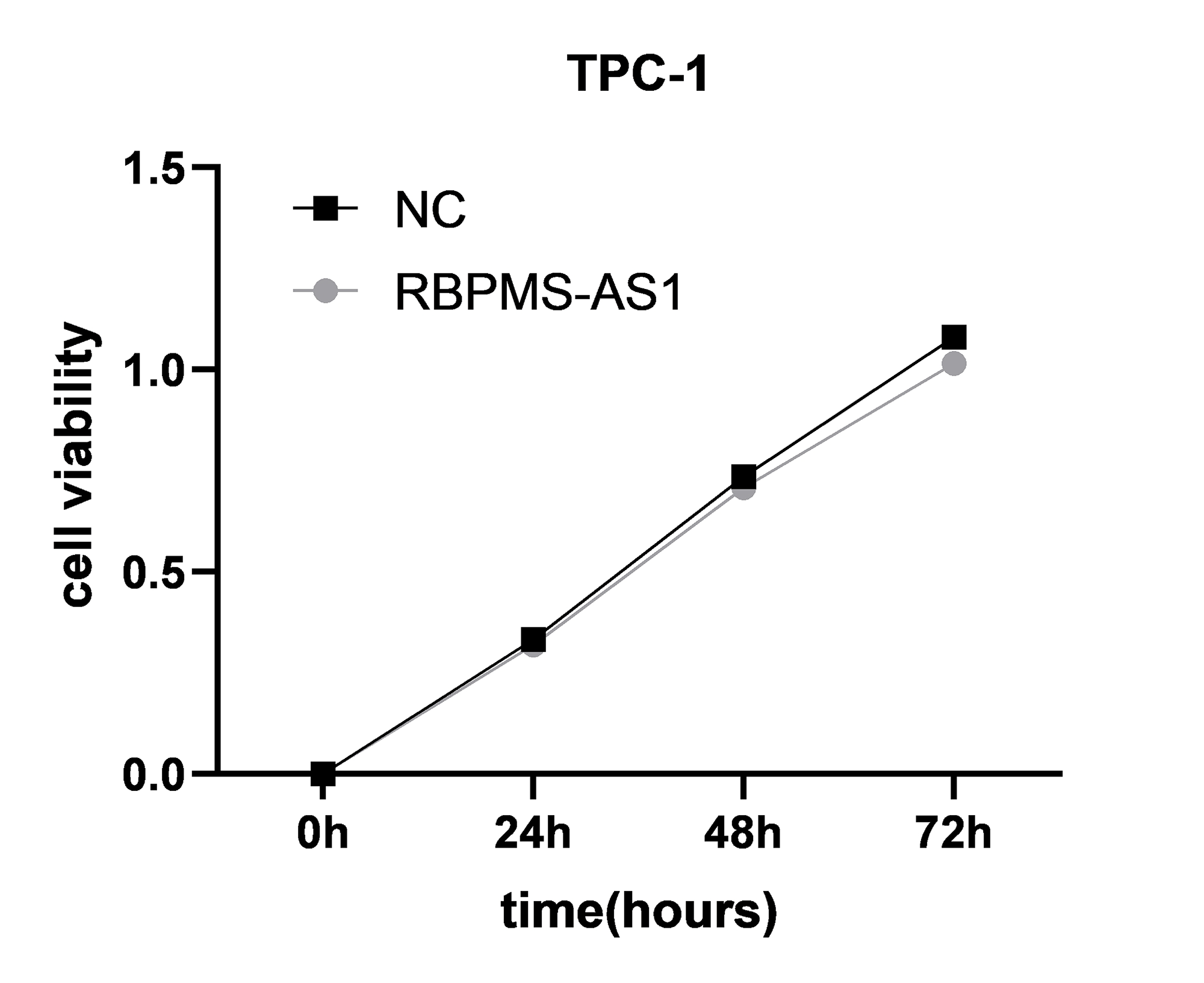

Supplement: Supplemental Information 3 [file peerj-11-15884-s003.png]

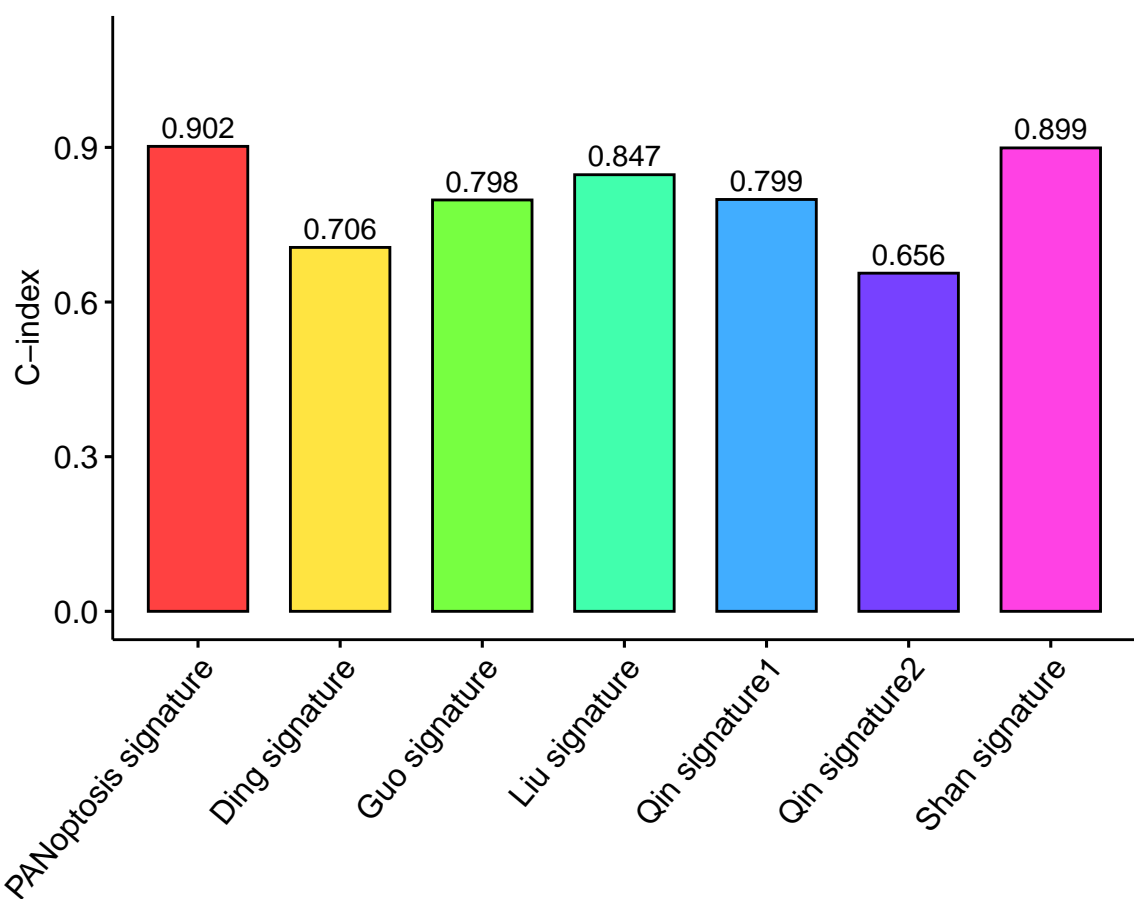

Supplement: Supplemental Information 9 [file peerj-11-15884-s009.zip › model comparison/C-index/C-index.pdf]

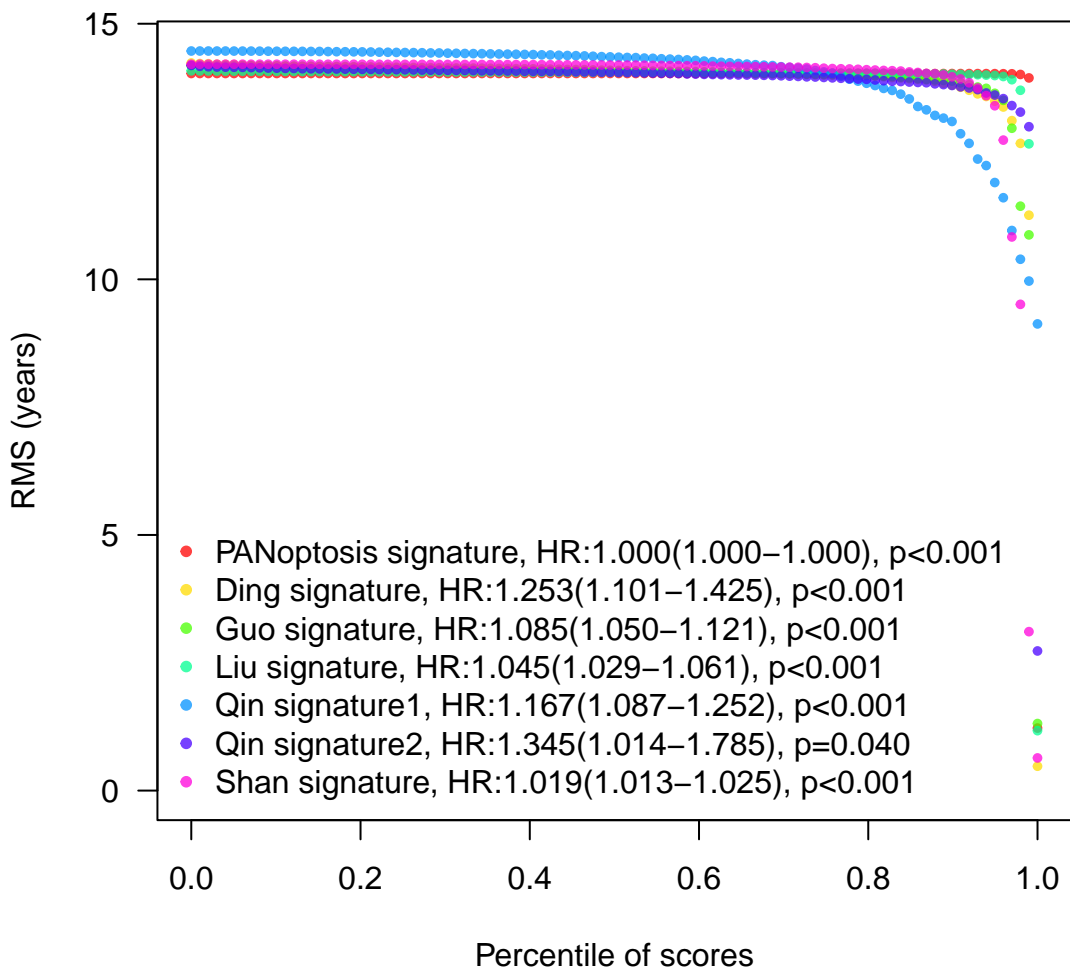

Supplement: Supplemental Information 9 [file peerj-11-15884-s009.zip › model comparison/C-index/RMS.pdf]

Sensitivity

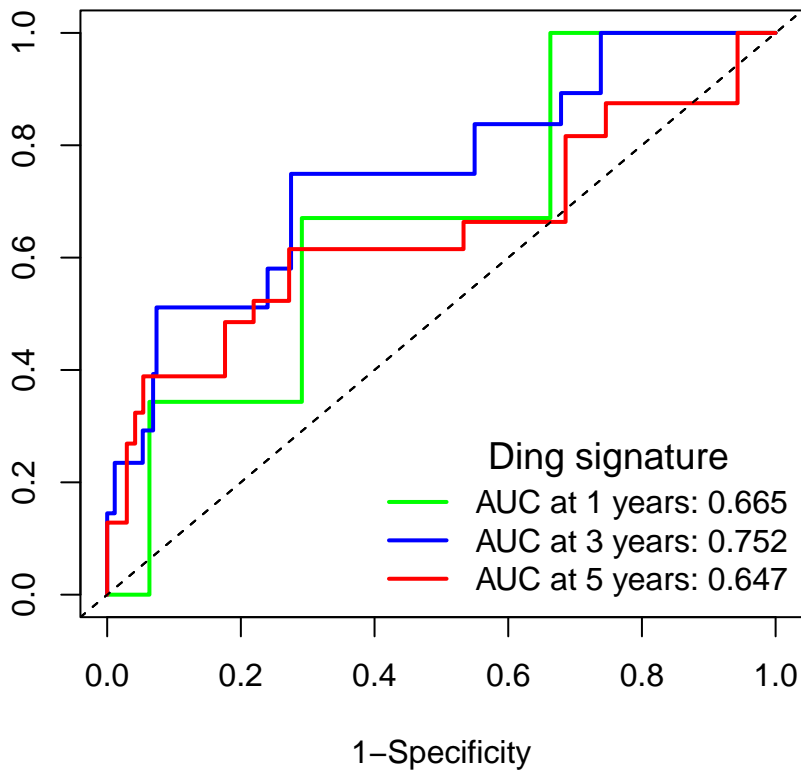

Supplement: Supplemental Information 9 [file peerj-11-15884-s009.zip › model comparison/ROC/ROC.Ding signature.pdf]

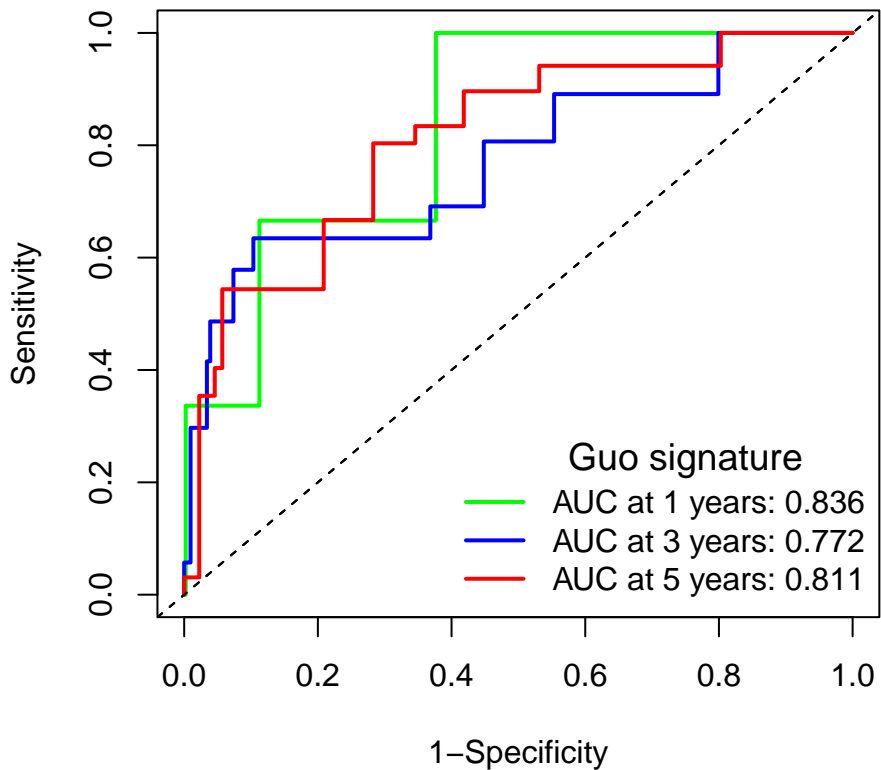

Supplement: Supplemental Information 9 [file peerj-11-15884-s009.zip › model comparison/ROC/ROC.Guo signature.pdf]

Sensitivity

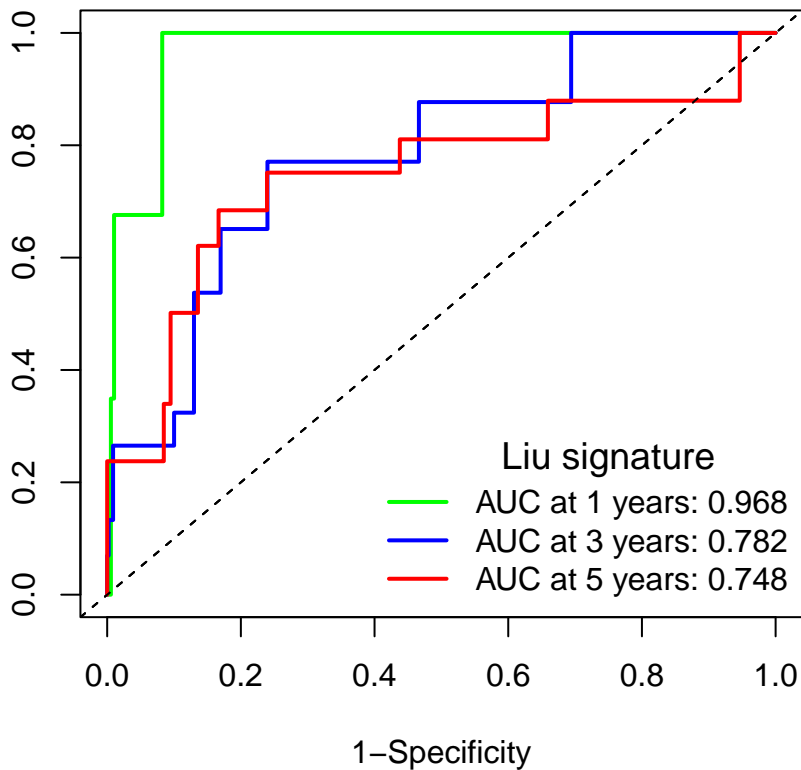

Supplement: Supplemental Information 9 [file peerj-11-15884-s009.zip › model comparison/ROC/ROC.Liu signature.pdf]

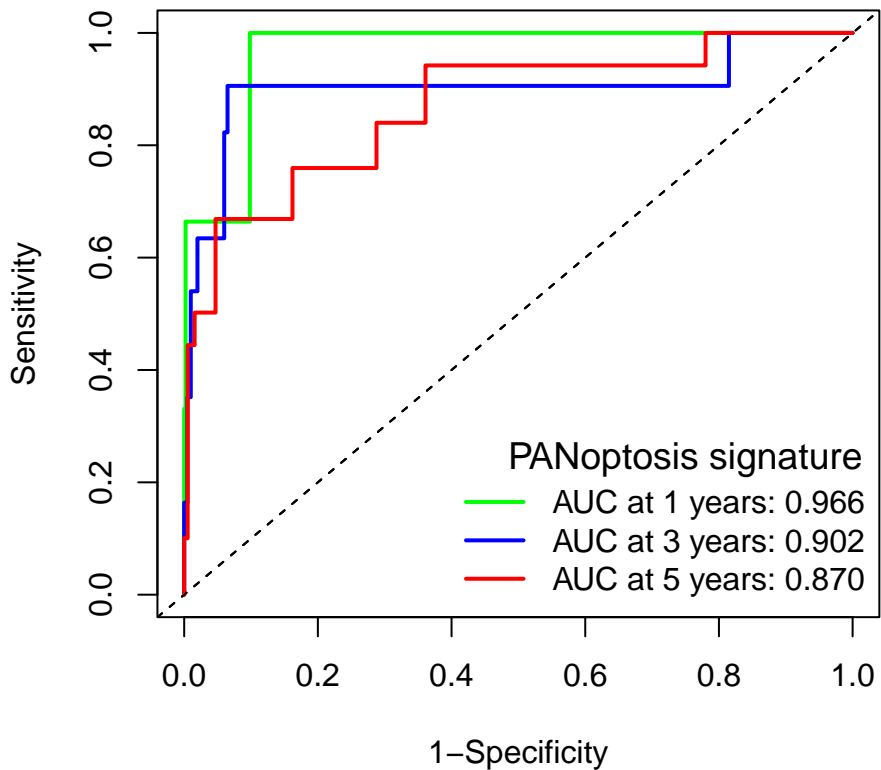

Supplement: Supplemental Information 9 [file peerj-11-15884-s009.zip › model comparison/ROC/ROC.PANoptosis signature.pdf]

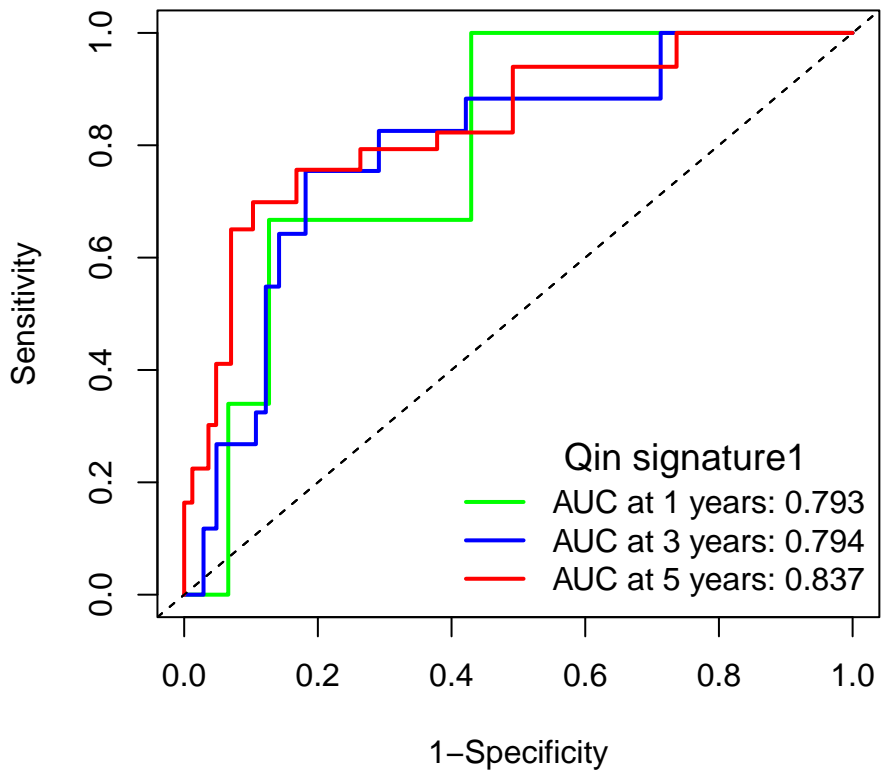

Supplement: Supplemental Information 9 [file peerj-11-15884-s009.zip › model comparison/ROC/ROC.Qin signature1.pdf]

Sensitivity

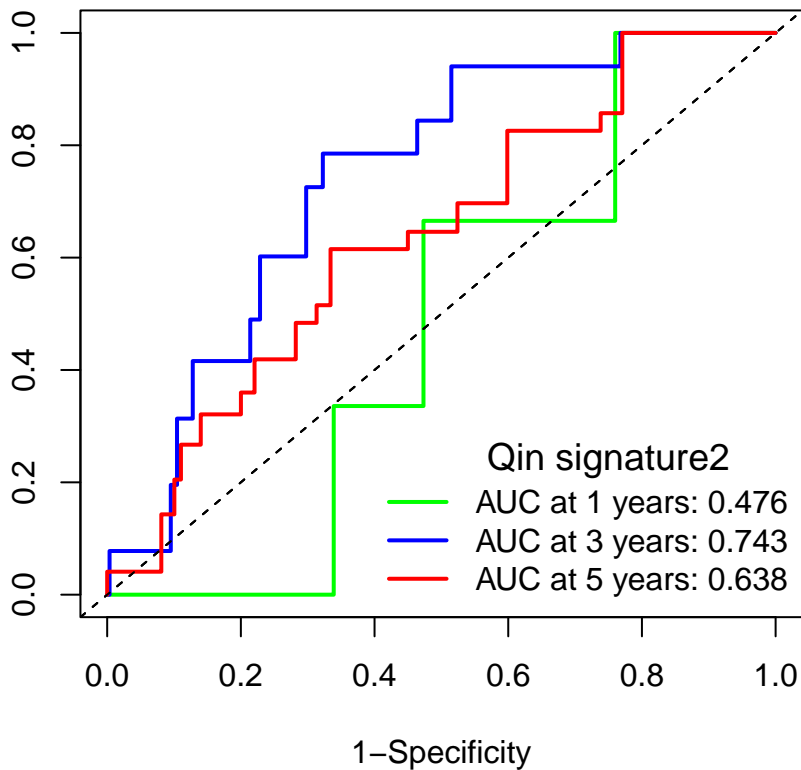

Supplement: Supplemental Information 9 [file peerj-11-15884-s009.zip › model comparison/ROC/ROC.Qin signature2.pdf]

Sensitivity

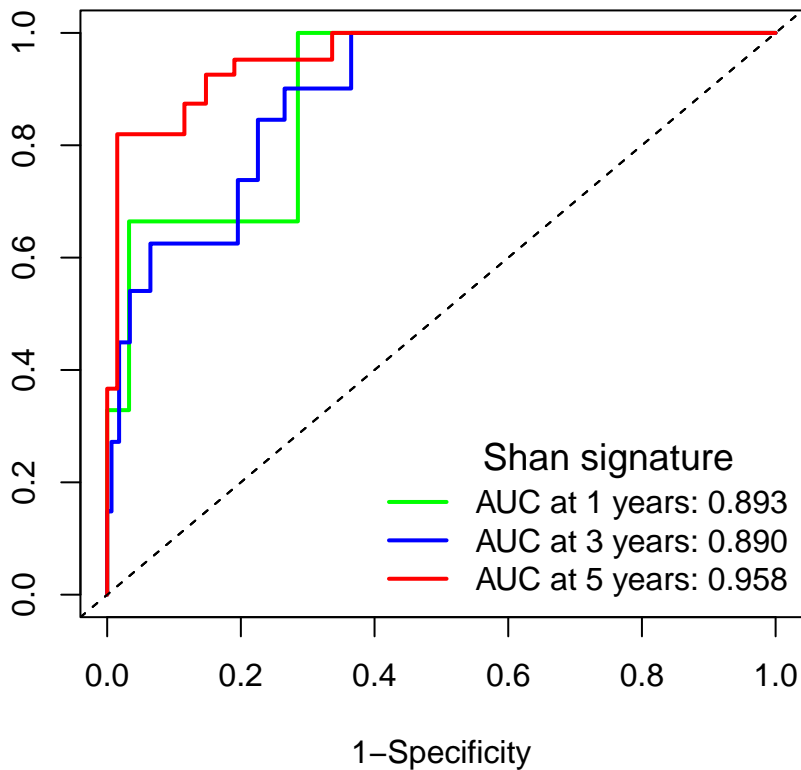

Supplement: Supplemental Information 9 [file peerj-11-15884-s009.zip › model comparison/ROC/ROC.Shan signature.pdf]

# Ding signature

Risk    + High risk    + Low risk

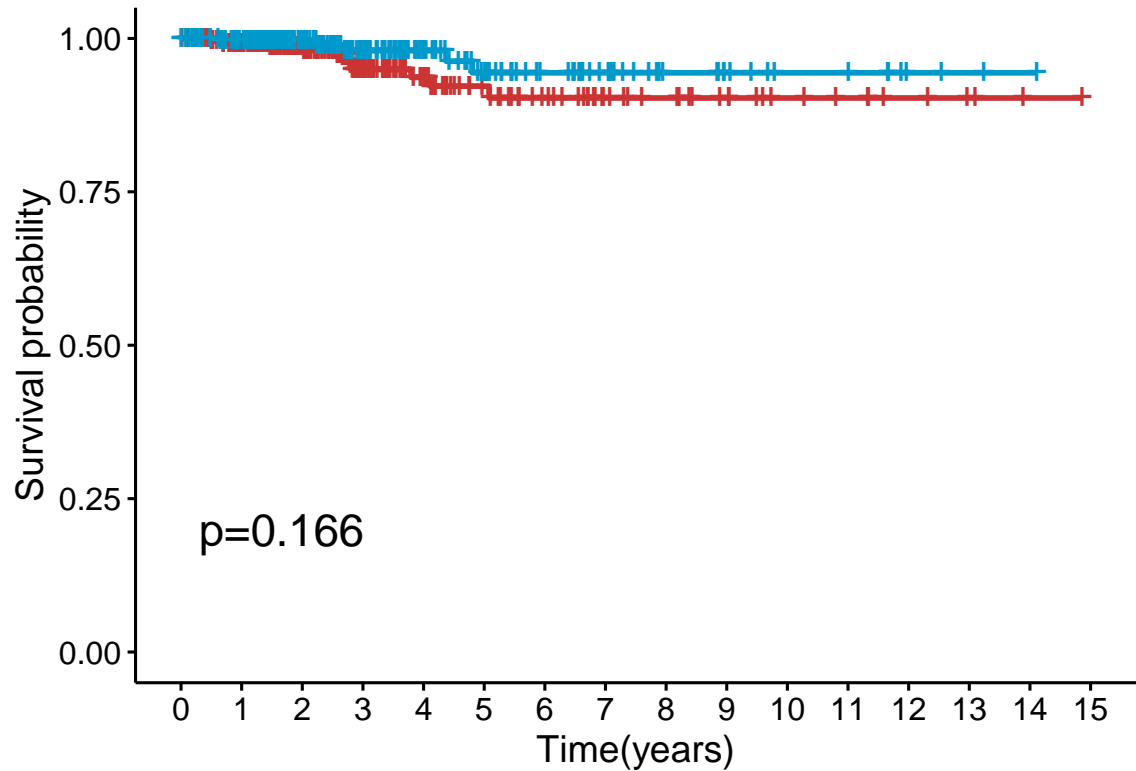

Supplement: Supplemental Information 9 [file peerj-11-15884-s009.zip › model comparison/ROC/sur.Ding signature.pdf]

# Guo signature

Risk    + High risk    + Low risk

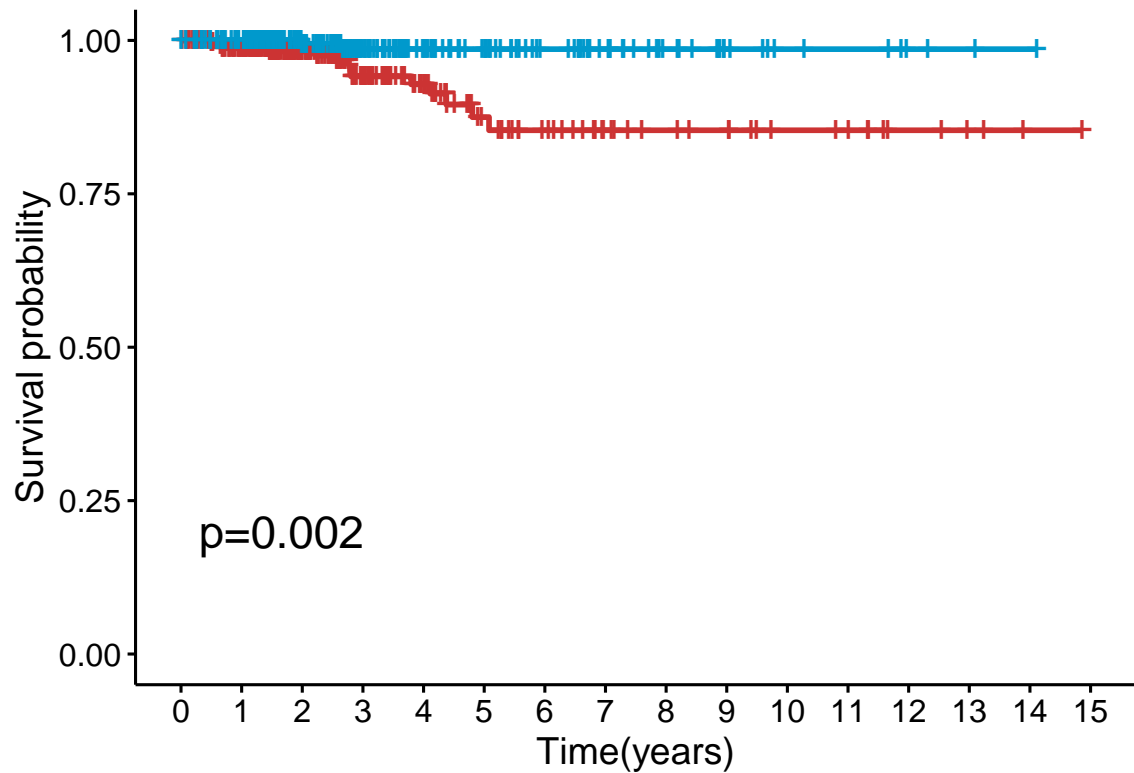

Supplement: Supplemental Information 9 [file peerj-11-15884-s009.zip › model comparison/ROC/sur.Guo signature.pdf]

# Liu signature

Risk    + High risk    + Low risk

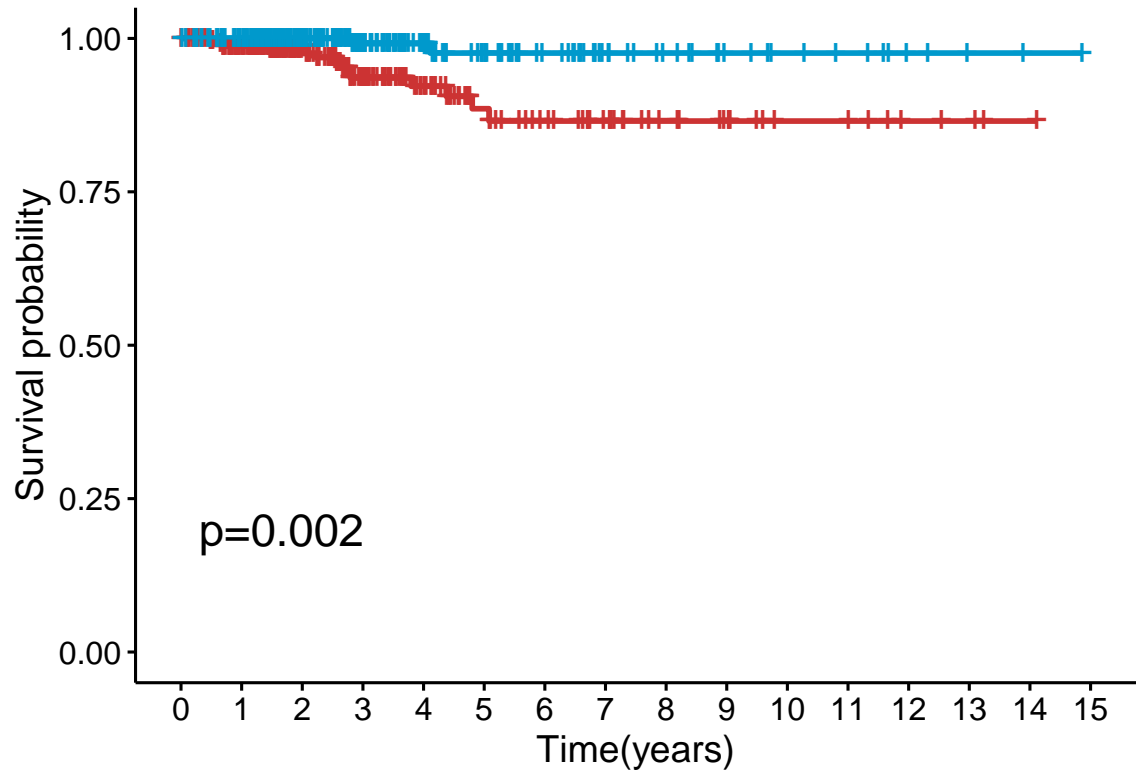

Supplement: Supplemental Information 9 [file peerj-11-15884-s009.zip › model comparison/ROC/sur.Liu signature.pdf]

# PANoptosis signature

Risk    + High risk    + Low risk

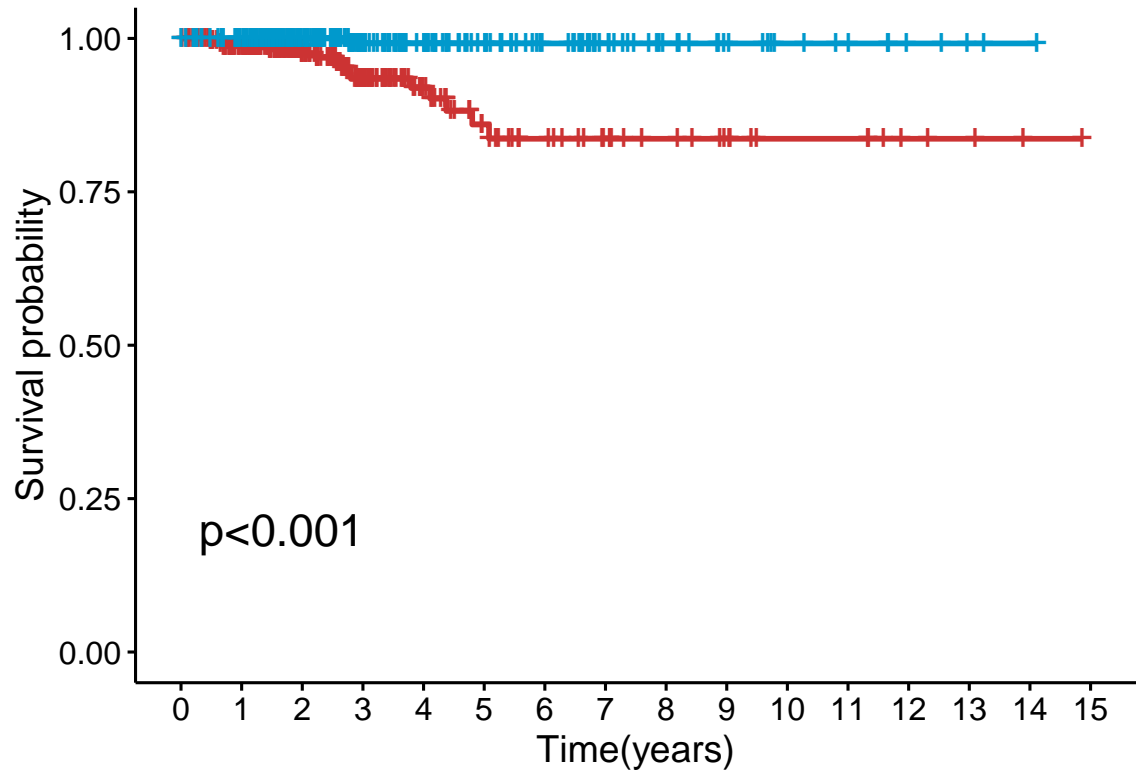

Supplement: Supplemental Information 9 [file peerj-11-15884-s009.zip › model comparison/ROC/sur.PANoptosis signature.pdf]

# Qin signature1

Risk    + High risk    + Low risk

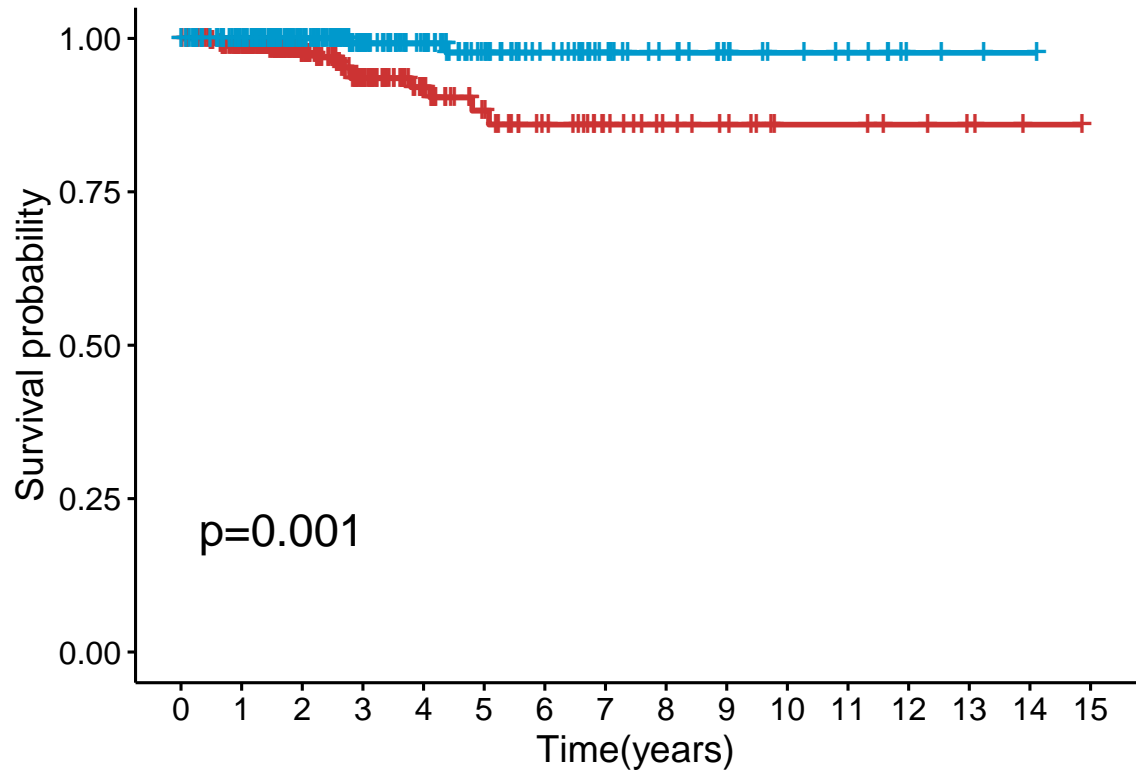

Supplement: Supplemental Information 9 [file peerj-11-15884-s009.zip › model comparison/ROC/sur.Qin signature1.pdf]

# Qin signature2

Risk    +    High risk    +    Low risk

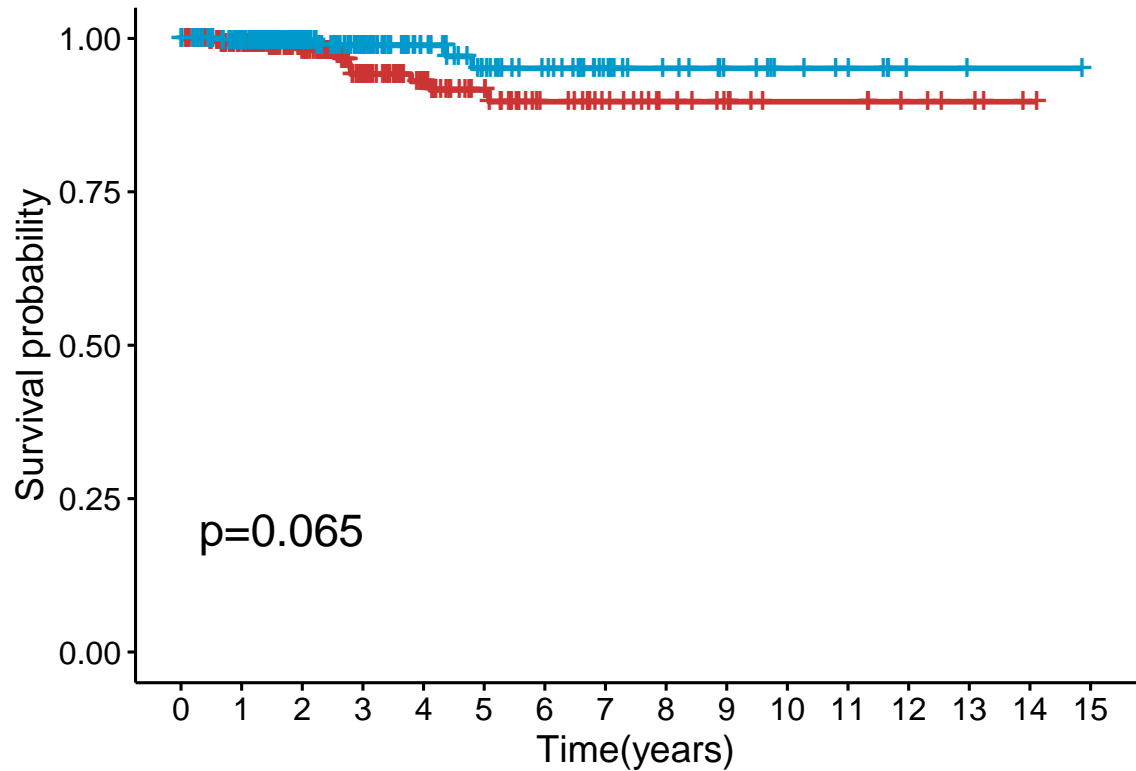

Supplement: Supplemental Information 9 [file peerj-11-15884-s009.zip › model comparison/ROC/sur.Qin signature2.pdf]

# Shan signature

Risk    + High risk    + Low risk

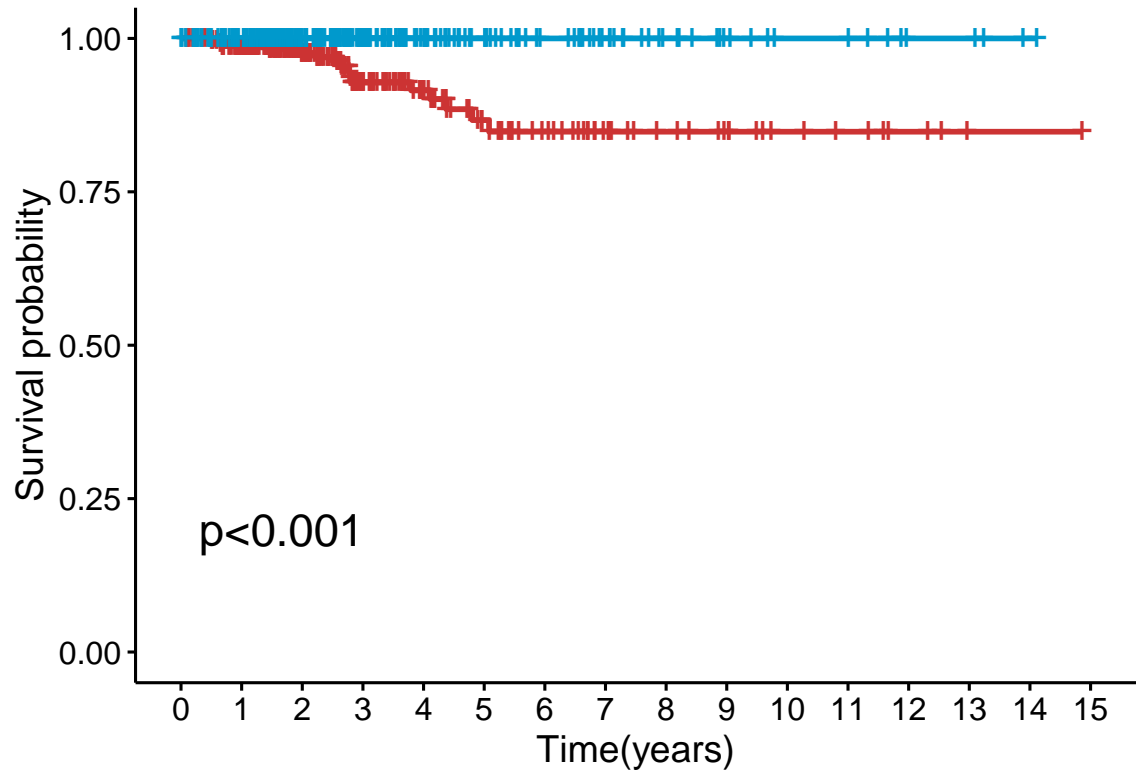

Supplement: Supplemental Information 9 [file peerj-11-15884-s009.zip › model comparison/ROC/sur.Shan signature.pdf]

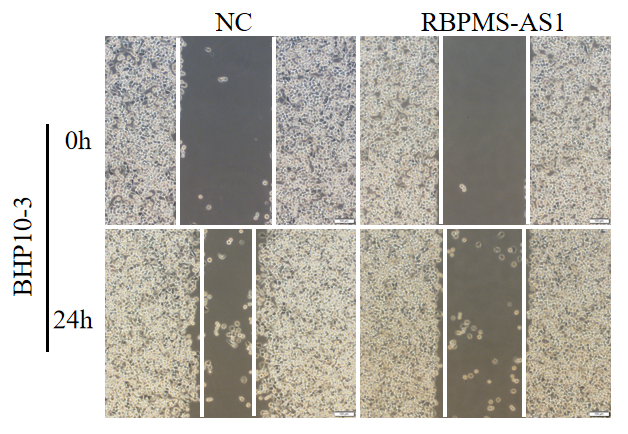

Supplement: Supplemental Information 10 [file peerj-11-15884-s010.zip › Wound healing/BHP10-3.png]

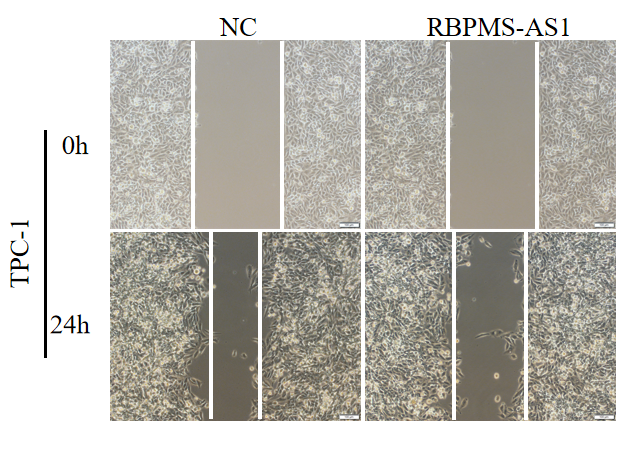

Supplement: Supplemental Information 10 [file peerj-11-15884-s010.zip › Wound healing/TPC-1.png]

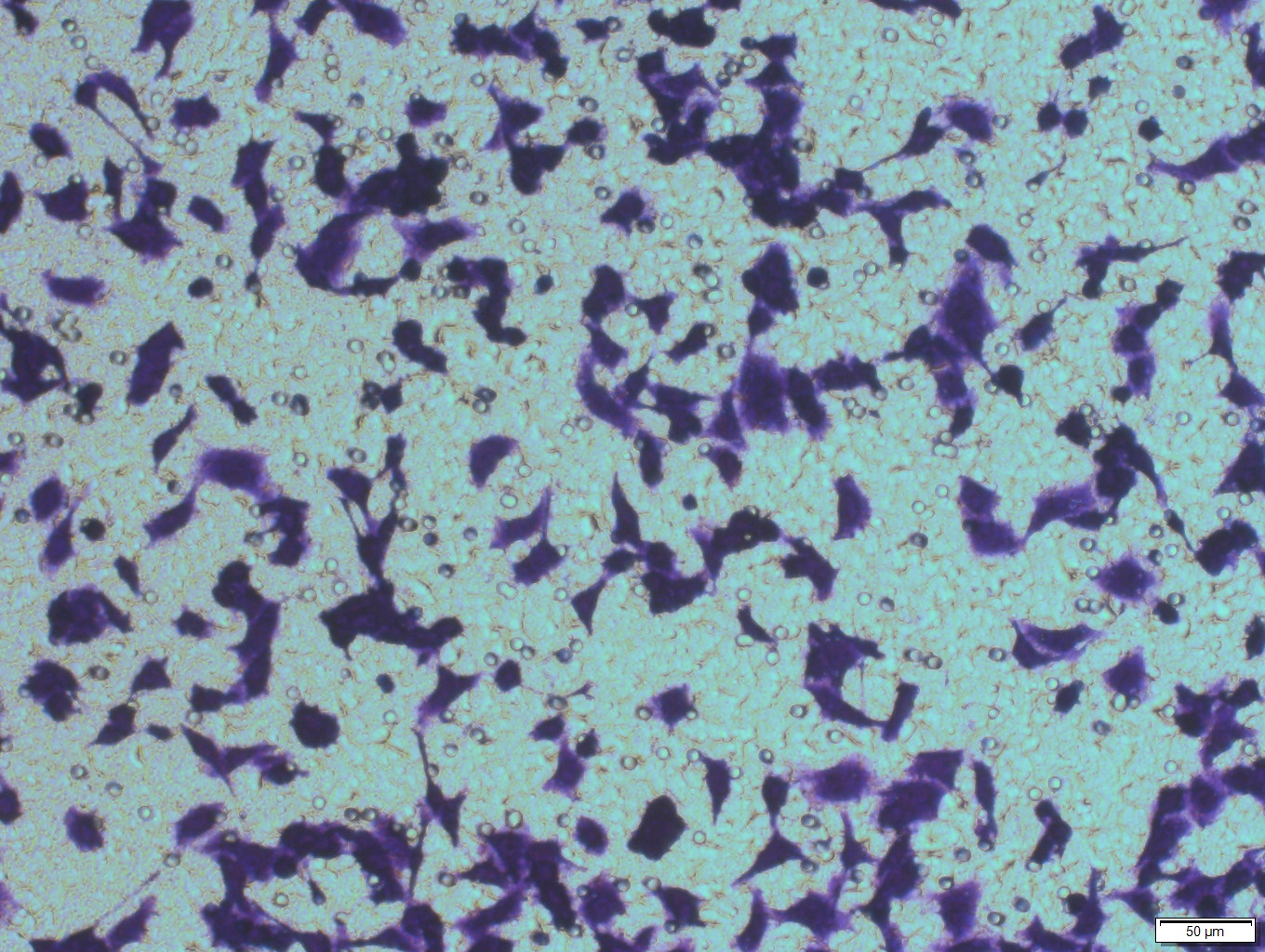

Supplement: Supplemental Information 11 [file peerj-11-15884-s011.zip › Migraton transwell/BHP10-3 NC.tif]

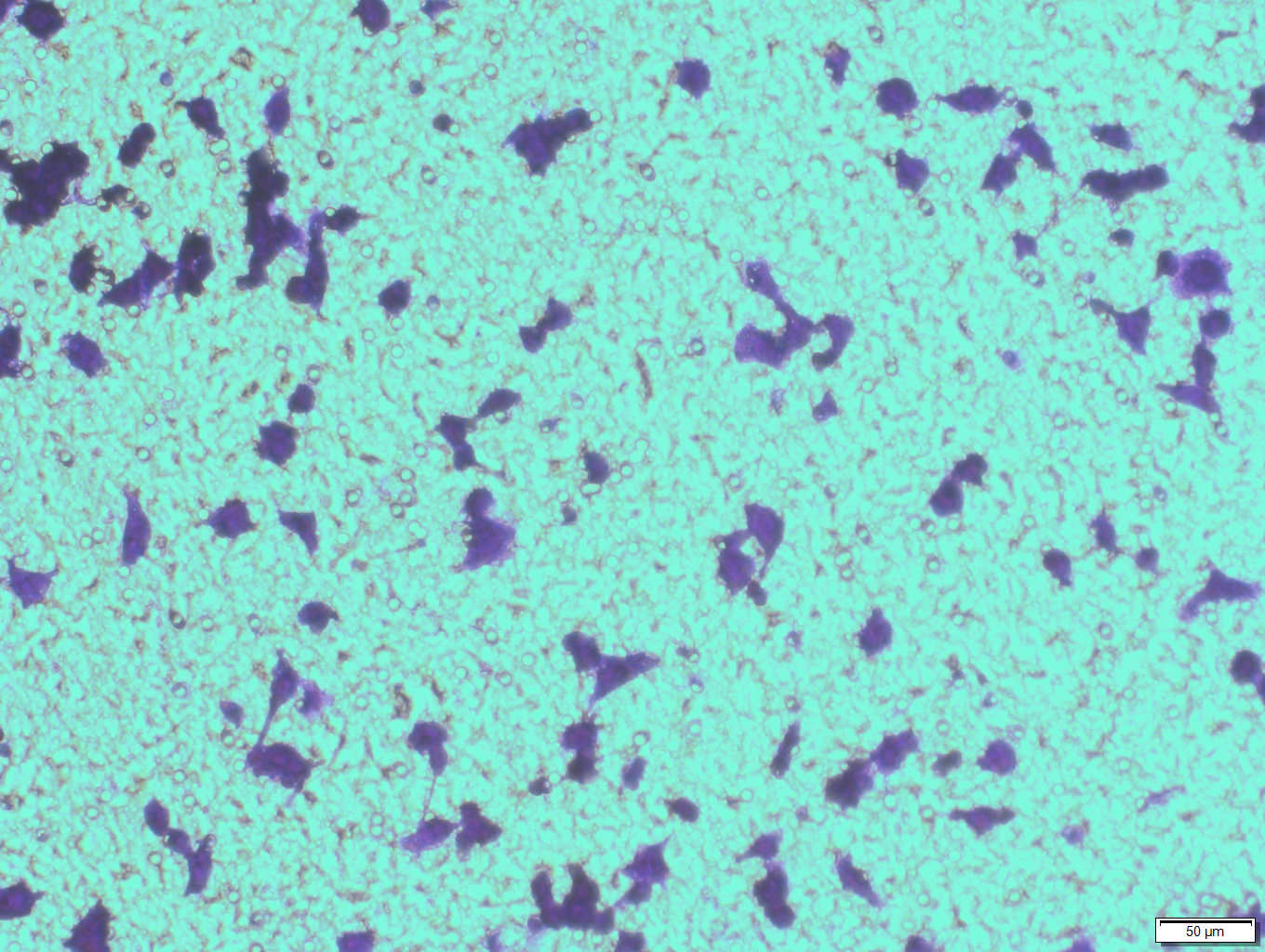

Supplement: Supplemental Information 11 [file peerj-11-15884-s011.zip › Migraton transwell/BHP10-3 RBPMS-AS1.tif]

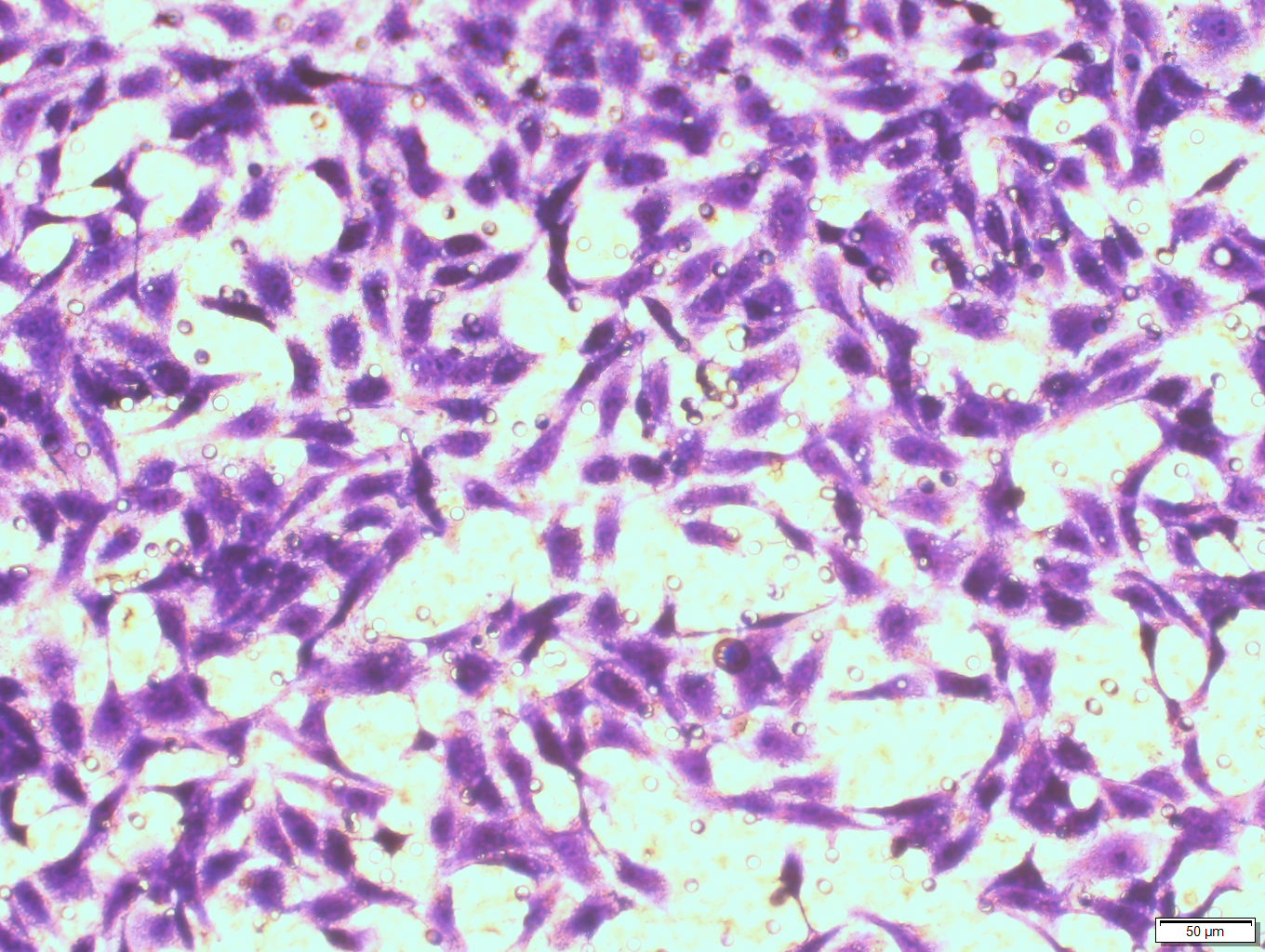

Supplement: Supplemental Information 11 [file peerj-11-15884-s011.zip › Migraton transwell/TPC-1 NC.tif]

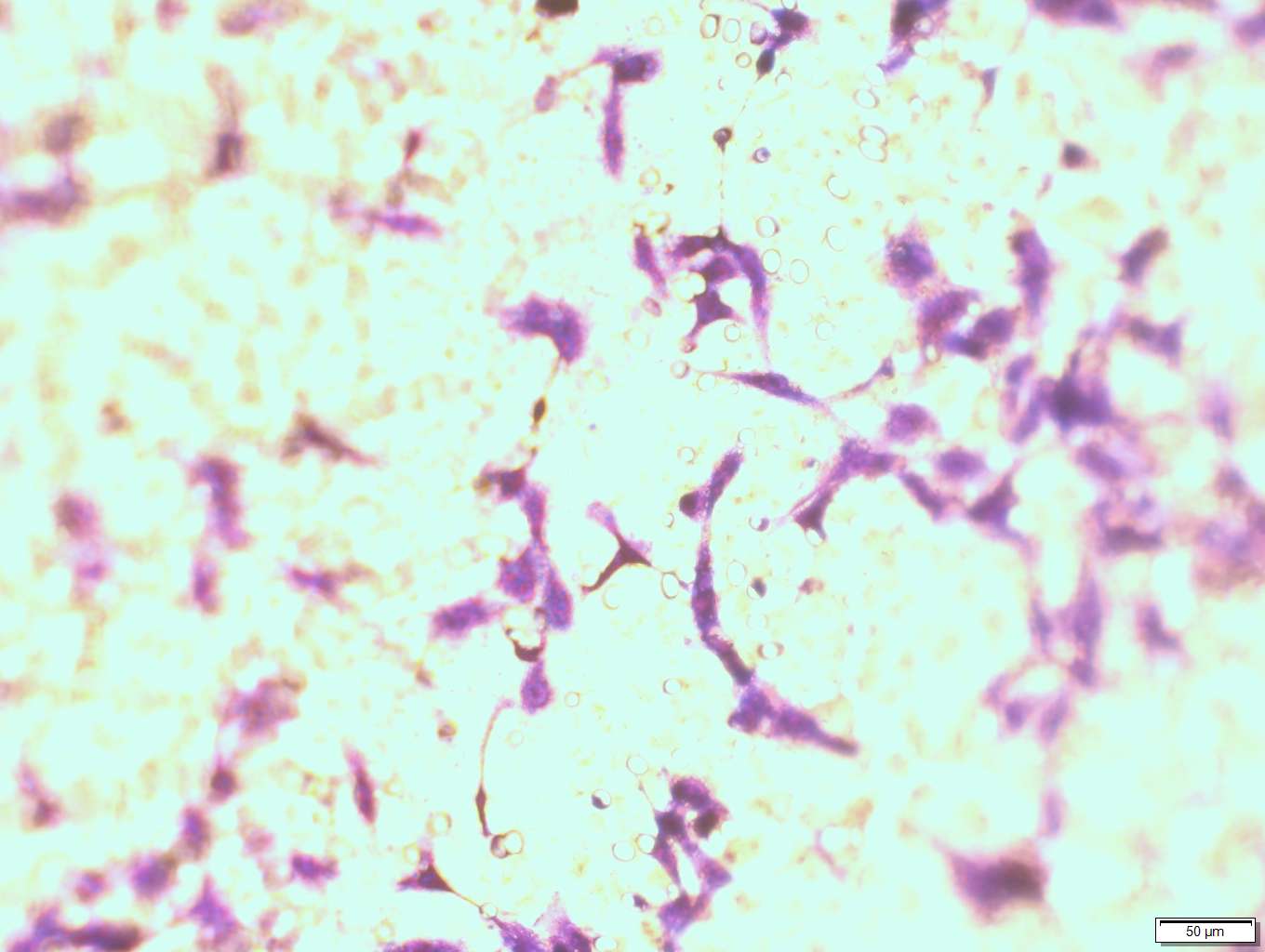

Supplement: Supplemental Information 11 [file peerj-11-15884-s011.zip › Migraton transwell/TPC-1 RBPMS-AS1.tif]

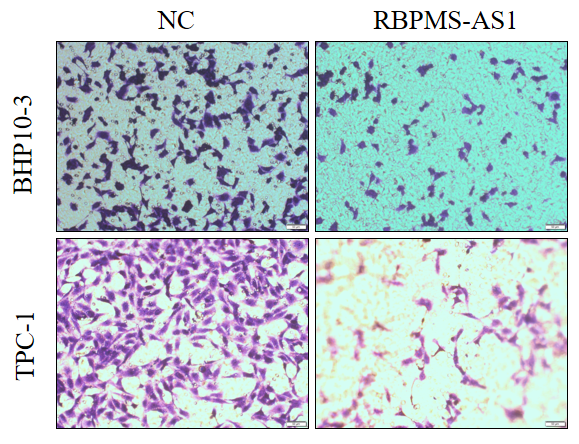

Supplement: Supplemental Information 11 [file peerj-11-15884-s011.zip › Migraton transwell/transwell.png]

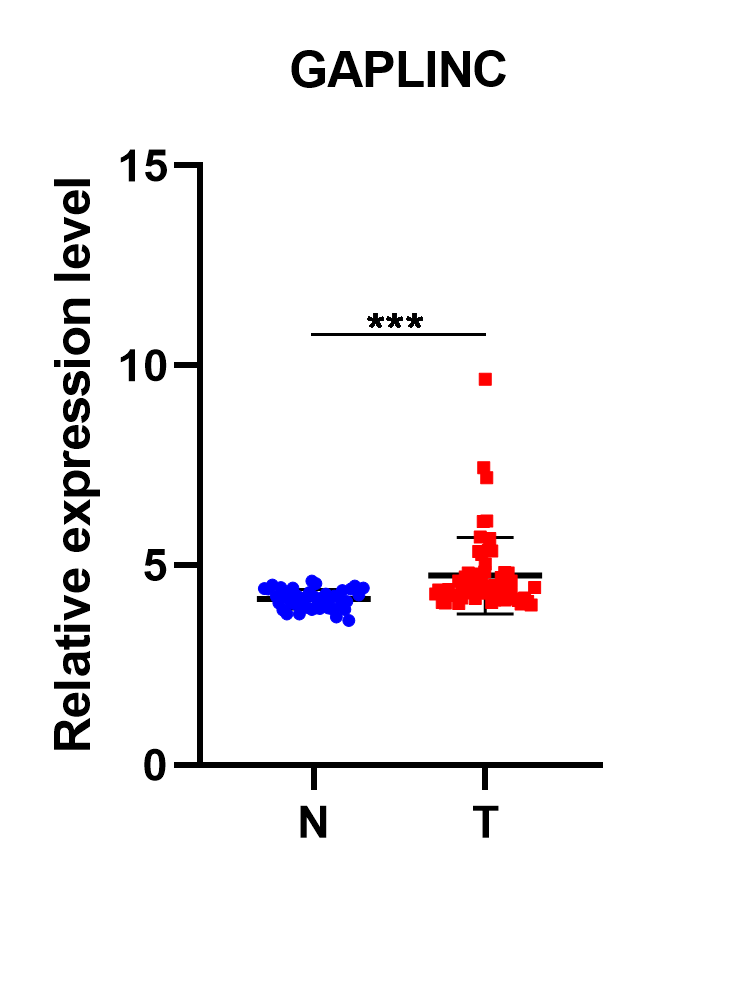

Supplement: Supplemental Information 12 [file peerj-11-15884-s012.zip › GSE33630/GSE33630 GAPLINC.png]

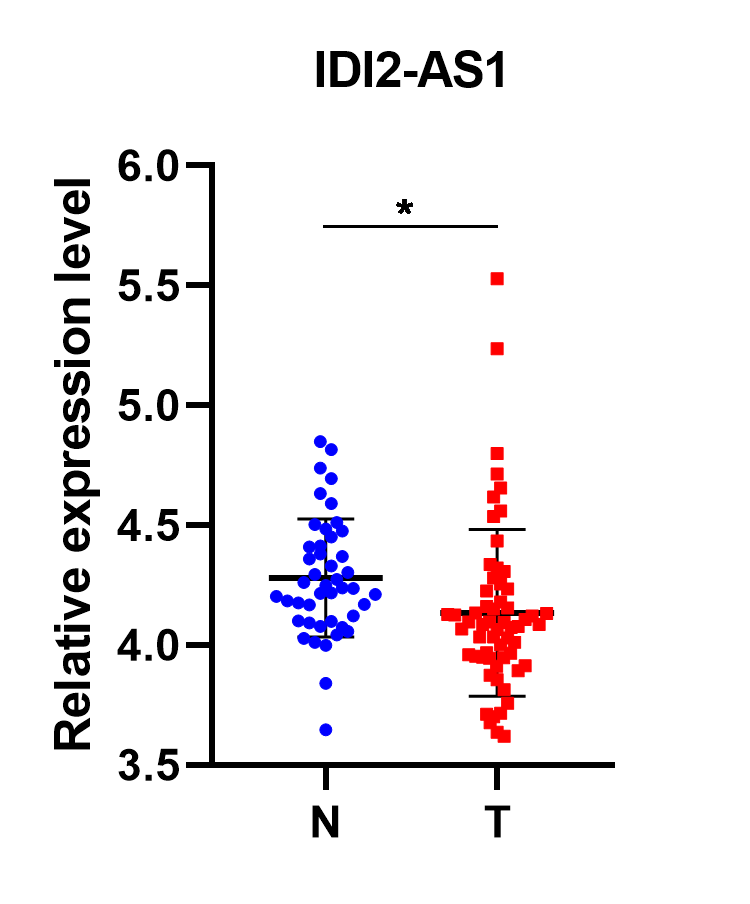

Supplement: Supplemental Information 12 [file peerj-11-15884-s012.zip › GSE33630/GSE33630 IDI2-AS1.png]

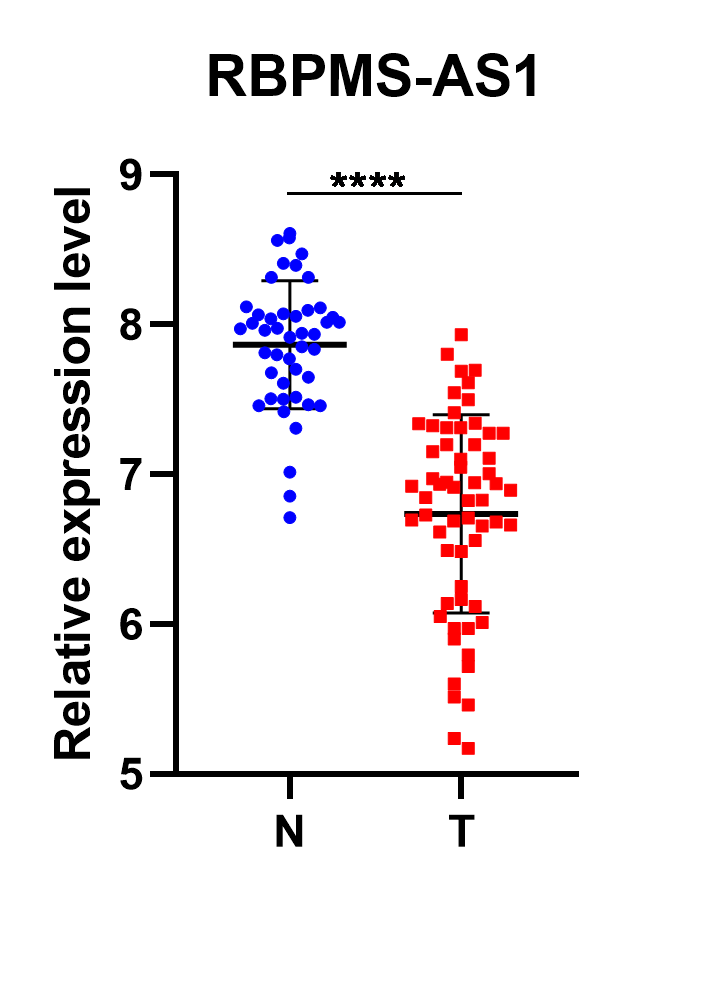

Supplement: Supplemental Information 12 [file peerj-11-15884-s012.zip › GSE33630/GSE33630 RBPMS-AS1.png]
